# Epigenome-Wide Association Study of Soluble Tumor Necrosis Factor Receptor 2 Levels in the Framingham Heart Study

**DOI:** 10.3389/fphar.2018.00207

**Published:** 2018-04-24

**Authors:** Michael M. Mendelson, Roby Johannes, Chunyu Liu, Tianxiao Huan, Chen Yao, Xiao Miao, Joanne M. Murabito, Josée Dupuis, Daniel Levy, Emelia J. Benjamin, Honghuang Lin

**Affiliations:** ^1^National Heart, Lung, and Blood Institute’s and Boston University’s Framingham Heart Study, Framingham, MA, United States; ^2^Department of Cardiology, Boston Children’s Hospital, Boston, MA, United States; ^3^Population Sciences Branch, Division of Intramural Research, National Heart, Lung, and Blood Institute, Bethesda, MD, United States; ^4^Hebrew SeniorLife, Harvard Medical School, Boston, MA, United States; ^5^Department of Biostatistics, Boston University School of Public Health, Boston, MA, United States; ^6^Innovation Research Institute of Traditional Chinese Medicine, Shanghai University of Traditional Chinese Medicine, Shanghai, China; ^7^Section of General Internal Medicine, Department of Medicine, Boston University School of Medicine, Boston, MA, United States; ^8^Section of Cardiovascular Medicine and Preventive Medicine, Department of Medicine, Boston University School of Medicine, Boston, MA, United States; ^9^Department of Epidemiology, Boston University School of Public Health, Boston, MA, United States; ^10^Section of Computational Biomedicine, Department of Medicine, Boston University School of Medicine, Boston, MA, United States

**Keywords:** DNA methylation, TNFR2 levels, inflammation, association studies, epigenome

## Abstract

**Background:** Transmembrane tumor necrosis factor (TNF) receptors are involved in inflammatory, apoptotic, and proliferative processes. In the bloodstream, soluble TNF receptor II (sTNFR2) can modify the inflammatory response of immune cells and is predictive of cardiovascular disease risk. We hypothesize that sTNFR2 is associated with epigenetic modifications of circulating leukocytes, which may relate to the pathophysiology underlying atherogenic risk.

**Methods:** We conducted an epigenome-wide association study of sTNFR2 levels in the Framingham Heart Study Offspring cohort (examination 8; 2005–2008). sTNFR2 was quantitated by enzyme immunoassay and DNA methylation by microarray. The concentration of sTNFR2 was log_e_-transformed and outliers were excluded. We conducted linear mixed effects models to test the association between sTNFR2 level and methylation at over 400,000 CpGs, adjusting for age, sex, BMI, smoking, imputed cell count, technical covariates, and accounting for familial relatedness.

**Results:** The study sample included 2468 participants (mean age: 67 ± 9 years, 52% women, mean sTNFR2 level 2661 ± 1078 pg/ml). After accounting for multiple testing, we identified 168 CpGs (*P* < 1.2 × 10^-7^) that were differentially methylated in relation to sTNFR2. A substantial proportion (27 CpGs; 16%) are in the major histocompatibility complex region and in loci overrepresented for antigen binding molecular functions (*P* = 1.7 × 10^-4^) and antigen processing and presentation biological processes (*P* = 1.3 × 10^-8^). Identified CpGs are enriched in active regulatory regions and associated with expression of 48 *cis*-genes (±500 kb) in whole blood (*P* < 1.1 × 10^-5^) that coincide with genes identified in GWAS of diseases of immune dysregulation (inflammatory bowel disease, type 1 diabetes, IgA nephropathy).

**Conclusion:** Differentially methylated loci in leukocytes associated with sTNF2 levels reside in active regulatory regions, are overrepresented in antigen processes, and are linked to inflammatory diseases.

## Introduction

Soluble tumor necrosis factor receptor II (sTNFR2) levels are associated with an extensive and diverse range of human diseases (Straczkowski et al., 2002; Turan et al., 2008; Doganavsargil-Baysal et al., 2013). In addition, sTNFR2 is a marker of cardiovascular disease (CVD) risk in people with diabetes (Shai et al., 2005) and chronic kidney disease (Neirynck et al., 2015). Tumor necrosis factor receptor II binds to tumor necrosis factors to form a heterocomplex that mediates an intricate inflammatory cascade. Released into the bloodstream, sTNFR2 can modify the inflammatory response of immune cells. A greater understanding of the changes that occur in circulating leukocytes in relation to sTNFR2 levels may lead to improved mechanistic insights, enhanced disease predictive modeling, and highlight novel candidates for the development of therapeutics for CVD and inflammatory-related diseases.

DNA methylation is an epigenetic modification that influences gene expression without changing the underlying genetic code. Adding a methyl group to the cytosine of cytosine-phosphate-guanine dinucleotides (CpGs) can modify the binding of transcription factors, influence the conformational structure of chromatin, and thus suppress the transcription of specific genes. Altered DNA methylation has been found to be linked to a variety of neoplastic, inflammatory, cardiometabolic, and atherosclerotic diseases (Ying et al., 2000; Maier and Olek, 2002; Fitzpatrick and Wilson, 2003; Hiltunen and Yla-Herttuala, 2003; Gallou-Kabani and Junien, 2005; Ling and Groop, 2009; Lund and Zaina, 2009).

In order to characterize the potential epigenetic link between sTNFR2 levels and disease, we conducted the first epigenome-wide association study of sTNFR2 levels with DNA methylation in circulating leukocytes from participants in a large community based cohort, the Framingham Heart Study (FHS). We also examined the association of sTNFR2-related differential methylation with gene expression in the same cohort. Finally, we integrated the findings with known biological pathways, genomic links to disease from genome-wide association studies, and targets of known drugs and compounds.

## Materials and Methods

### Study Sample

The FHS is a longitudinal community-based cohort, started in 1948. Three generations of participants have been recruited. The present study was focused on participants from the second generation, the Offspring cohort (Kannel et al., 1979) who attended the eighth examination between 2005 and 2008. We excluded participants with self-reported auto-immune diseases, such as rheumatoid arthritis, or who took immune modulating agents (e.g., methotrexate), steroids, and synthetic estrogens. All participants gave written informed consent and the study was approved by the Institutional Review Board of Boston Medical Center.

### Measurement of sTNFR2 Levels in Plasma

Fasting morning blood samples were obtained during the routine clinic visit. The samples were then frozen at -80°C before processing. The sTNFR2 levels were measured by the quantitative enzyme-linked immunosorbent assay per the manufacturers’ protocols (R&D Systems, Minneapolis, MN, United States). The assay shows high reproducibility rate with intra-assay coefficients of variation of 2.3% (Jefferson et al., 2007). The sTNFR2 levels were natural log transformed and values outside of four standard deviation of the mean were excluded from the analysis (*n* = 1).

### DNA Methylation Quantification

DNA from whole blood samples used for methylation assays was collected at the same examination assessment as the sTNFR2 levels, anthropometric and covariate measurements. Buffy coat preparations were obtained from peripheral whole blood samples and DNA was extracted using the Gentra Puregene DNA extraction kit (Qiagen, Venlo, Netherlands) and then underwent bisulfite conversion using the EZ DNA Methylation kit (Zymo Research, Irvine, CA, United States). Samples underwent whole genome amplification, fragmentation, array hybridization, and single-base pair extension. DNA methylation arrays were run in two laboratory batches at the Johns Hopkins Center for Inherited Disease Research and University of Minnesota Biomedical Genomics Center. The first batch included 576 samples from an earlier CVD case-control study (Joehanes et al., 2013) and the second batch included 2270 samples from the remainder of the offspring cohort participants. DNA methylation results underwent normalization within laboratory batches using the DASEN methodology implemented in the wateRmelon package (Pidsley et al., 2013) in R (version 3.0.2). We excluded samples with missing rate > 1%, poor single nucleotide polymorphism (SNP) matching to the 65 SNP control probe locations, and outliers by multi-dimensional scaling techniques. We also excluded probes with missing rate > 20% and those previously identified to map to multiple locations (Chen et al., 2013) or to have an underlying SNP (minor allele frequency > 5% in European participants from the 1000 Genomes Project) at the CpG site, or within 10 bp of the single base extension (*n* = 42,251). The methylation data are available at dbGaP under the accession number phs000724.v2.p9.

### Gene Expression Assay

The details of gene expression profiling were previously described (Joehanes et al., 2013). In brief, gene expression was obtained from whole blood samples collected in PAXgene blood tubes (PreAnalytiX, Hombrechtikon, Switzerland) at the same time as DNA methylation samples. RNA was extracted using a whole blood RNA System Kit (QIAGEN, Hilden, Germany) and assayed using the Affymetrix Human Exon Array ST-1.0 (Affymetrix, Santa Clara, CA, United States). Robust Multi-array Average (RMA) package in R was used to normalize gene expression values. Residuals were obtained from linear regression models after adjustment for technical covariates and imputed differential blood cell proportions. The *pedigreemm* package was used to remove the variation due to age, sex, and family structure. The gene expression data is available at dbGaP under the accession number phs000363.v16.p10.

### Clinical Covariate Assessment

Height and weight were measured using established protocols and body mass index (BMI) was calculated as weight (in kilograms) divided by height (in meters) squared. Self-reported cigarette smoking was categorized based on current smoking status (current smoker, non-current smoker).

### Statistical Analyses

#### Epigenome-Wide Association Study of sTNFR2

We used linear mixed effects regression models, whereas the untransformed beta value of methylation at the CpG site was treated as the dependent variable, and the sTNFR2 level was treated as the independent variable, adjusted for age, sex, BMI, smoking status, and technical covariates. The sex was coded as “1” for men and “2” for women. For the smoking status, we coded current smokers as “1” and the rest of participants as “0.” Technical covariates included known batch effects (methylation array, row, and column) and two methylation principal components to account for unmeasured batch effects. To account for the family relatedness in Framingham, a covariance matrix was calculated based on the pedigree, and was used as the random effect for the linear mixed effects models. We additionally adjusted for the proportion of six cell types imputed via a reference panel (Houseman method) (Houseman et al., 2012), including CD8^+^ T cells, CD4^+^ T cell, natural killer cells, B cells, monocytes and granulocytes. Bonferroni correction was used to adjust for multiple testing, and significant CpG sites were defined as those with *P* < 0.05/N, where N was the number of tests. The analyses were performed using the “lmekin” function within R package “kinship”, and the clinical and technical covariates were used as fixed effect in the models.

#### Association of sTNFR2-Related CpG Methylation With Gene Expression

Each epigenome-wide significant CpG from the sTNFR2 EWAS analysis was tested using linear mixed effect models for the association with expression levels of genes in *cis* (±500 kb). The *cis* expression-methylation association models specified the gene expression residual as the dependent variable, the DNA methylation residual as the independent variable of interest and additionally adjusted for 25 methylation surrogate variables and 25 expression surrogate variables to account for unmeasured technical and batch effects. Multiple testing was accounted for by Bonferroni correction for the number of CpGs in distinct loci (>500 kb) tested times the number of unique *cis* genes on the expression array.

#### Enrichment Analysis

We used eFORGE (Breeze et al., 2016) (v1.2)^1^ to examine if sTNFR2-related CpG sites are enriched in DNAse I hypersensitivity hotspots (markers of active regulatory regions) and loci with overlapping histone modifications (H3Kme1, H3Kme4, H3K9me3, H3K27me3, and H3K36me3) across cell lines and tissues from the Roadmap Epigenome, BLUEPRINT Epigenome, and ENCODE consortia data (Adams et al., 2012; ENCODE Project Consortium et al., 2012; Roadmap Epigenomics Consortium et al., 2015).

We used the PANTHER Overrepresentation Test^2^ (release 20171205) to identify whether identified gene loci are overrepresented in specific molecular function and biological process gene ontologies (slim version) with Fisher’s Exact test and false discovery rate (FDR) multiple testing correction. We then used FUMA (Watanabe et al., 2017) (v1.2.4)^3^ to examine whether CpG-related differentially expressed genes are enriched for specific diseases identified in genome-wide association studies (GWAS) from GWAS catalog version e85 (2016.09.27), restricting to at least three overlapping genes, and FDR multiple testing correction.

## Results

The study sample, outlined in **Table 1**, included 2,468 FHS participants (mean age 67 ± 9 years, 52% women) from the eighth examination cycle (2005–2008). The distribution of sTNFR2 levels is shown in Supplementary Figure 1 (mean level 2661 ± 1078 pg/ml).

**Table 1:** Clinical characteristics of studied samples.

Characteristics	Total (*n* = 2468)
Women, *n* (%)	1282 (51.9%)
Age, year ±*SD*	67 ± 9
sTNFR2 levels, pg/ml	2661 ± 1078
Cigarette smoking, n (%)	217 (8.8%)
Body mass index, kg/m^2^	28.3 ± 5.3

### Association of Methylation Profiles With sTNFR2 Levels

**FIGURE 1:**
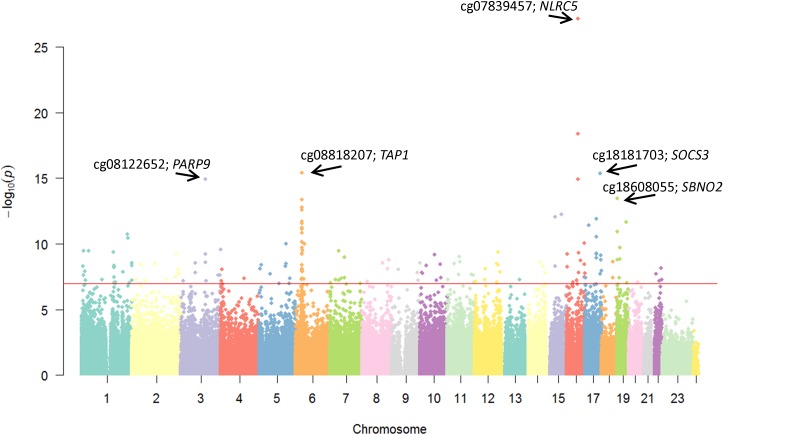
Manhattan plot of methylation of CpG sites associated with sTNFR2 levels. The annotations for the top five loci are shown.

We found methylation at 168 CpG sites to be associated at epigenome-wide thresholds (*P* < 1.2 × 10^-7^) with sTNFR2 levels (complete list in Supplementary Table 1) after adjustment for age, sex, BMI, smoking, imputed cell counts, methylation principal components, technical covariates, and accounting for familial relatedness. **Figure 1** depicts the Manhattan plot of CpG sites associated with sTNFR2 with the top CpG sites noted in **Table 2**. The strongest association was at cg07839457 (*P* = 3.2 × 10^-22^), located 436 bp upstream of the transcription start site of *NLRC5*, which is involved in cytokine response, inhibition of NF-κB activation and negative regulation of type I interferon signaling pathways (Cui et al., 2010).

**Table 2:** Top 20 CpG sites associated with sTNFR2 levels.

CPG	Chr	Position (hg19)	Closest gene	Effect	*SE*	*P*-value
cg07839457	16	57,023,022	*NLRC5*	-0.0520	0.0047	6.7 × 10^-28^
cg16411857	16	57,023,191	*NLRC5*	-0.0246	0.0027	4.3 × 10^-19^
cg08818207	6	32,820,355	*TAP1*	-0.0201	0.0025	3.9 × 10^-16^
cg18181703	17	76,354,621	*SOCS3*	-0.0217	0.0026	4.1 × 10^-16^
cg08159663	16	57,022,486	*NLRC5*	-0.0148	0.0018	1.1 × 10^-15^
cg08122652	3	122,281,939	*PARP9*	-0.0196	0.0024	1.2 × 10^-15^
cg18608055	19	1,130,866	*SBNO2*	-0.0156	0.0020	3.4 × 10^-14^
cg08099136	6	32,811,251	*PSMB8*	-0.0195	0.0026	4.4 × 10^-14^
cg01309328	6	32,811,253	*PSMB8*	-0.0170	0.0023	1.7 × 10^-13^
cg07333021	6	30,612,330	*C6orf134*	0.0133	0.0018	2.6 × 10^-13^
cg23387863	15	77,472,416	*SGK269*	-0.0147	0.0020	5.4 × 10^-13^
cg22107533	15	45,028,083	*TRIM69*	-0.0172	0.0024	9.1 × 10^-13^
cg16936953	17	57,915,665	*TMEM49*	-0.0251	0.0035	1.2 × 10^-12^
cg11187245	6	31,323,397	*HLA-B*	-0.0209	0.0030	2.0 × 10^-12^
cg26470501	19	45,252,955	*BCL3*	-0.0149	0.0021	2.2 × 10^-12^
cg16186435	6	32,810,833	*PSMB8*	-0.0140	0.0020	2.7 × 10^-12^
cg02656560	17	19,967,600	*SPECC1*	-0.0114	0.0016	3.7 × 10^-12^
cg13031097	6	31,322,577	*HLA-B*	-0.0195	0.0028	6.3 × 10^-12^
cg23533285	6	31,322,348	*HLA-B*	-0.0180	0.0026	7.4 × 10^-12^
cg07573872	19	1,126,342	*SBNO2*	-0.0192	0.0028	1.2 × 10^-11^

*SE, standard error.*

#### Functional and Regulatory Annotation of sTNFR2-Related CpG Sites

The 168 sTNFR2-related differentially methylated CpGs are enriched in active regulatory regions (DHS hotspots) across most blood cell lines (especially inflammatory macrophages, *P* < 1 × 10^-15^), but also vascular and cardiac tissues (*P* < 1 × 10^-4^) (Supplementary Figure 2). Many sTNFR2-related CpGs are located within histone modification regions; primarily H3K4me1 (marker of enhancers), and to a lesser extent H3K4me3 (marker of promoters) and H3K36me3 (found in actively transcribed gene bodies) in blood cell lines (Supplementary Table 3). Unsurprisingly, a large proportion of sTNFR2-related CpG sites are located within the major histocompatibility complex (MHC) region (27 CpGs; 16%). Other top loci were in gene regions known to be involved in NF-κB and cytokine pathways, such as *NLRC5*, *SOCS3*, *BCL3* and *SBNO2*. sTNFR2-related loci are overrepresented in antigen binding molecular functions (*P* = 1.7 × 10^-4^, FDR = 3.2 × 10^-2^) and antigen processing and presentation biological processes (*P* = 1.3 × 10^-8^, FDR = 3.1 × 10^-6^).

### Association of sTNFR2-Related CpG Sites With Gene Expression

We also examined if the sTNFR2-related CpG sites are associated with gene expression in whole blood. The 168 CpG sites are in 127 distinct loci, comprising 4352 genes in *cis* (±500 kb). As shown in Supplementary Table 2, these CpGs were associated with the expression of 27 genes after adjustment for multiple testing (*P* < 1.1 × 10^-5^). Over 5% of the variation in expression (partial r^2^) was explained by methylation at the identified CpGs for three genes (*HCP5, AIM2, and NMUR1*). *HCP5* is a non-coding gene located in the MHC class I region, and is linked to viral interactions, neoplastic progression, and psoriatic arthritis (Liu et al., 2008). The expression of *AIM2* is induced by interferon gamma and plays a role in the innate immune system. *NMUR1* is a transmembrane protein involved in signal transduction.

We then assessed the disease relevance of the 27 genes that demonstrated differential expression with sTNFR2-related CpG sites. **Table 3** shows the top enriched gene sets. Many of them are involved in immune dysregulation (inflammatory bowel disease, type 1 diabetes, and autoimmune thyroid disease), clotting factors (fibrinogen), and obesity-related traits. A query of the DrugBank database (version 5.0.9) (Law et al., 2014) showed that six of these genes were targets of known drugs or compounds, including *PSMB8* (carfilzomib), S100P (chromolyn sodium), TYMP (tipiracil, cidofovir, trifuridine, fluorouracil, floxuridine, and capecitabine), *GLTP* (capric acid, lauric acid, oleic acid, and sphingosine), *SLC22A4* and *SLC22A5* (by numerous drugs that influence transmembrane channels).

**Table 3:** Enrichment of identified genes in genome-wide association studies of human disease.

Gene set	Number of genes in the set	Number of overlapping genes	*P*-value	Adjusted *P*-value	Overlapping genes
Type 1 diabetes and autoimmune thyroid diseases	23	3	4.4 × 10^-9^	4.8 × 10^-6^	*HCP5, PRRC2A, TAP2*
Fibrinogen	41	3	5.0 × 10^-8^	1.8 × 10^-5^	*CPT1B, SLC22A4, SLC22A5*
Ulcerative colitis	447	5	2.4 × 10^-6^	3.2 × 10^-4^	*LSP1, SLC22A4, SLC22A5, TCF19, PRRC2A*
Inflammatory bowel disease	719	5	3.5 × 10^-5^	2.2 × 10^-3^	*LSP1, SLC22A4, SLC22A5, TCF19, PRRC2A*
Obesity-related traits	745	4	4.6 × 10^-4^	1.2 × 10^-2^	*IFI16, AIM2, ODF3B, S100P*
Blood protein levels	914	4	1.2 × 10^-3^	2.4 × 10^-2^	*HCP5, PRRC2A, TAP2, TAP2, PSMB8*
Crohn’s disease	612	3	2.0 × 10^-3^	3.6 × 10^-2^	*SLC22A4, SLC22A5, HLA-F*

## Discussion

We present the first epigenome-wide analysis of leukocyte-derived DNA methylation in relation to sTNFR2 levels in a large community-based cohort. We identified differential methylation at 168 CpGs within 127 distinct genomic loci that are enriched for active regulatory regions across inflammatory macrophages and vascular tissues. Our results highlight functional relevance of genomic regulation of antigen presentation and processing in relation to sTNFR2 levels with multiple loci identified in the MHC region. Differentially methylated loci are associated with gene expression of 27 *cis*-genes in whole blood that have clinical relevance for inflammatory diseases, clotting factors, and obesity-related traits. Taken together, delineation of genomic regulatory alterations in relation to sTNFR2 demonstrates a host of altered pathways that may be important targets for novel or known therapeutics to prevent CVD and other sTNFR2-related diseases.

The most significant CpG site resides within the promoter region of *NLRC5.* The *NLRC5* gene encodes a nucleotide binding protein that belongs to the highly conserved NOD-like protein family, which regulates multiple interferon signaling pathways (Cui et al., 2010). *NLRC5* has been shown to be cardioprotective; deficiency of *NLRC5* exacerbates high fat diet-induced heart injury in murine models (Ma and Xie, 2017). Another significant sTNFR2-related CpG site, cg08818207, is in the first intron of *TAP1*, which encodes a transporter belonging to the ATP binding cassette subfamily. The protein is involved in the transportation of a variety of molecules across the cell membranes. It is essential to the presentation of peptides to human leukocyte antigen molecules. Mutations in *TAP1* result in immune-related diseases such as MHC class I deficiency (Hanalioglu et al., 2017) but are also associated with cardiometabolic diseases, such as type 2 diabetes (Li et al., 2014).

As expected, among genes that showed differential expression with sTNFR2-related CpG sites, many of them are involved in inflammatory and autoimmune diseases. One example is *SLC22A4*, which encodes an organic cation transporter that plays an essential role in the elimination of small organic cations such as environmental toxins and drugs (Morrissey et al., 2013; Nigam et al., 2015). It has already been proposed as potential drug target for the treatment of inflammatory diseases such as rheumatoid arthritis (Tokuhiro et al., 2003; Martinez et al., 2006; Maeda et al., 2007) and Crohn’s disease (Leung et al., 2006). An additional five identified genes are targets of known drugs or compounds. For example, carfilzomib, an anti-neoplastic and selective proteasome inhibitor, influences PSMB8, proteasome subunit beta 8. Carfilzomib has already been shown to have cardiovascular effects with substantial cardiotoxicity observed in clinical oncology trials (Waxman et al., 2017).

Our study has limitations. First, unlike genetic sequence variants, differences in DNA methylation may occur secondary to the phenotype of interest and not causal effects. Despite not being able to determine the direction of effect, secondary changes may still be important for downstream consequences, such as CVD. Second, current study measured DNA methylation profiles in the whole blood, which contains a mixture of different types of cells. Although we adjusted for cell proportion changes using statistical approaches, residual confounding due to unmeasured cell type differences or other unmeasured technical or clinical factors may be present. Third, although we assayed over four hundred thousand CpGs, there are many unmeasured CpGs sites and therefore additional methylation differences may be relevant. Fourth, we only assessed DNA methylation from leukocytes and did not assay DNA methylation concurrently in other tissues in the body where findings may differ. Therefore, our assessment is focused on circulating immune-cell related changes. Fifth, as sTNFR2 is not a widely measured biomarker, we were unable to replicate our findings in an external cohort with DNA methylation. Further studies are needed to determine if our findings replicate in other cohorts and are generalizable to other age groups and ethnicities/races.

## Conclusion

EWAS of leukocyte DNA for CVD-related inflammatory biomarkers represents a powerful approach to gain novel insights into affected biological pathways. In our sTNFR2 EWAS, we highlight antigen processing pathways and specific genes in NF-κB signaling and cytokine development and release. Genes in these pathways have previously been linked to human disease GWAS of inflammatory diseases, clotting factors, and obesity-related traits. Further experimental studies and trials are needed to determine if perturbation of these genes will support the repurposing of known drugs or development of novel compounds which may prevent or treat CVD and other sTNFR2 related diseases.

## Author Contributions

MMM and HL drafted the manuscript. MMM, RJ, CL, TH, CY, and HL performed the analyses. XM, JMM, JD, DL, and EJB critically reviewed the manuscript.

## Conflict of Interest Statement

The authors declare that the research was conducted in the absence of any commercial or financial relationships that could be construed as a potential conflict of interest.

## Footnotes

1 eforge.cs.ucl.ac.uk

2 www.pantherdb.org

3 fuma.ctglab.nl
