# Supplementary material for: Epigenome-Wide Association Study of Soluble Tumor Necrosis Factor Receptor 2 Levels in the Framingham Heart Study
Source: Front Pharmacol. 2018 Apr 24;9:207. doi: 10.3389/fphar.2018.00207 (PMC5928448; doi:10.3389/fphar.2018.00207)

Supplemental Figure 1. Distribution of sTNFR2 levels in the Framingham Heart Study Offspring cohort participants (examination 8; 2005-2008)

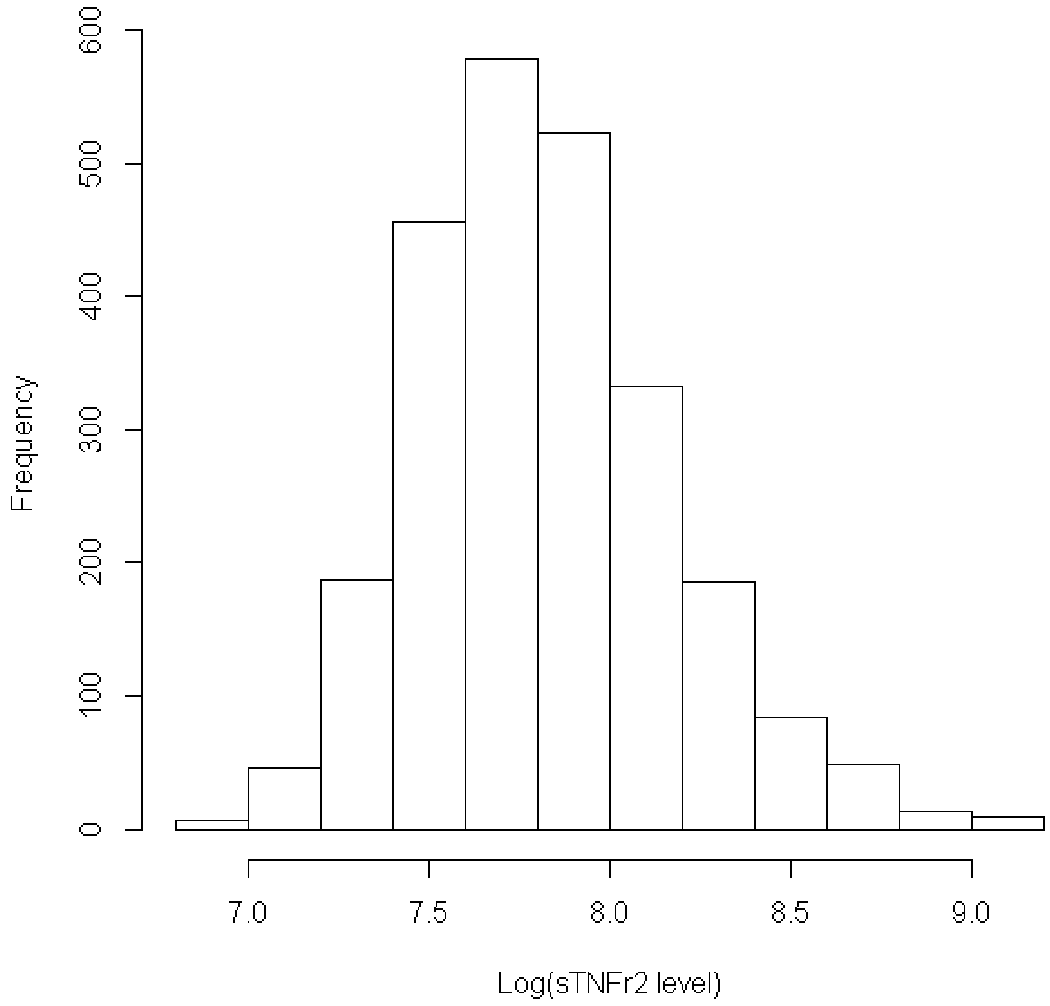

Supplemental Figure 2. Enrichment of sTNFR2-related CpG sites in regulatory regions

Consolidated Roadmap Epigenomics – DNAse Hypersensitivity Sites

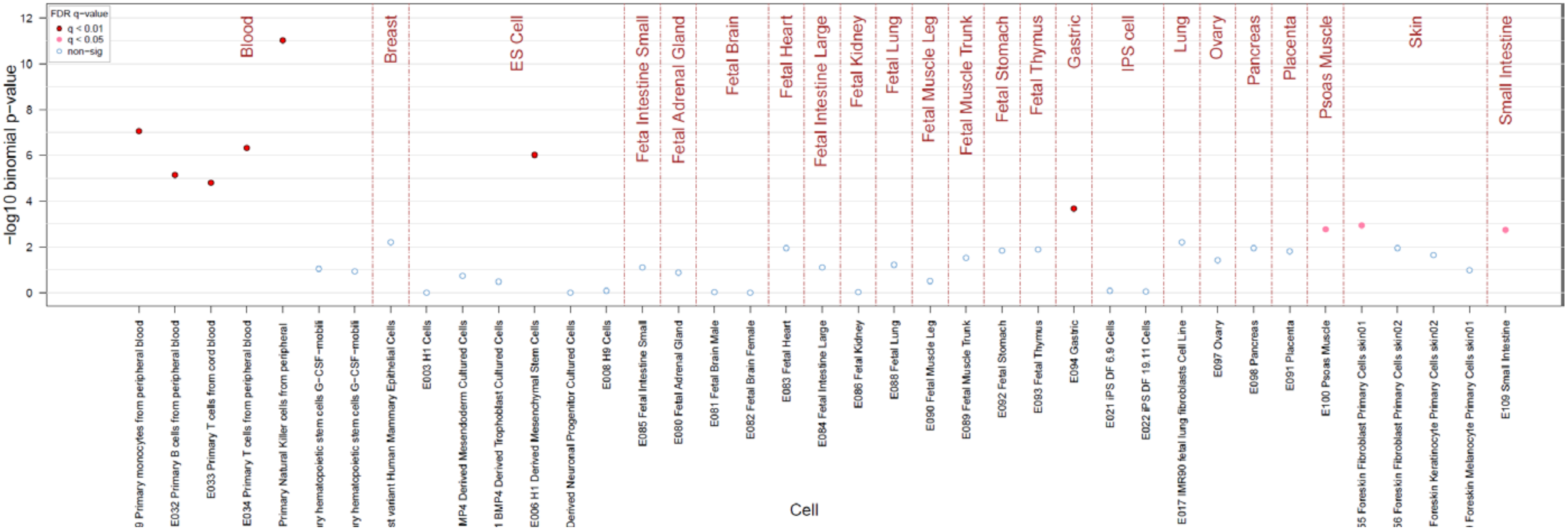

Roadmap Epigenomics (2012) – DNAse Hypersensitivity Sites

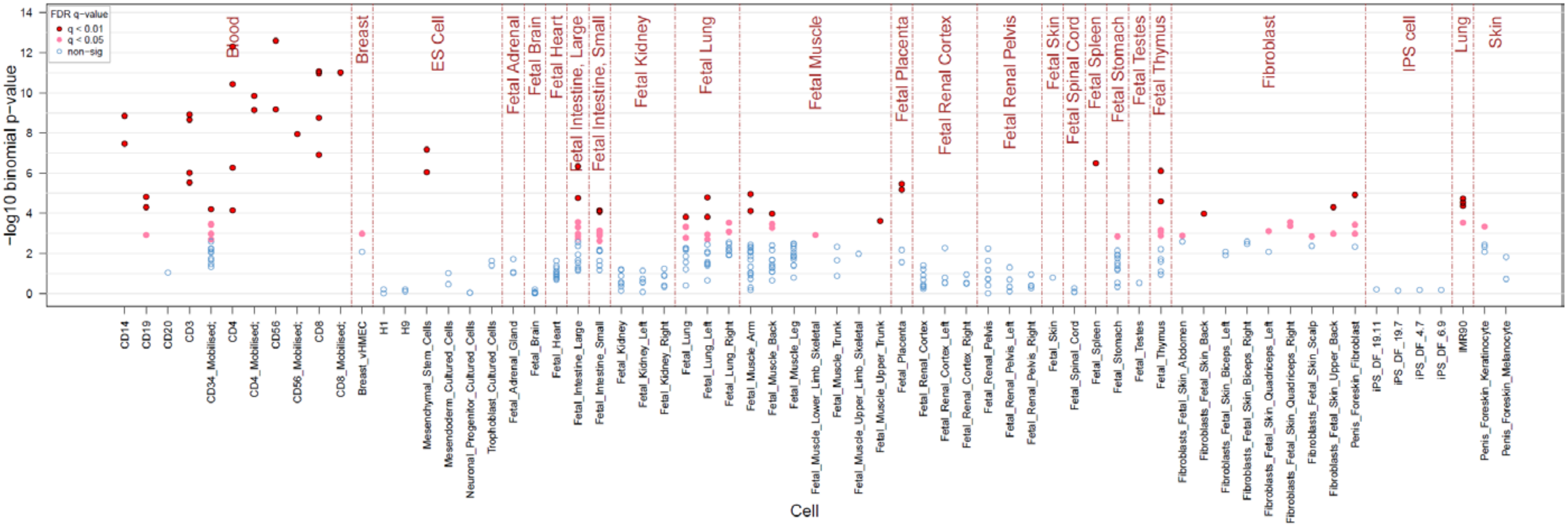

ENCODE – DNAse Hypersensitivity Sites

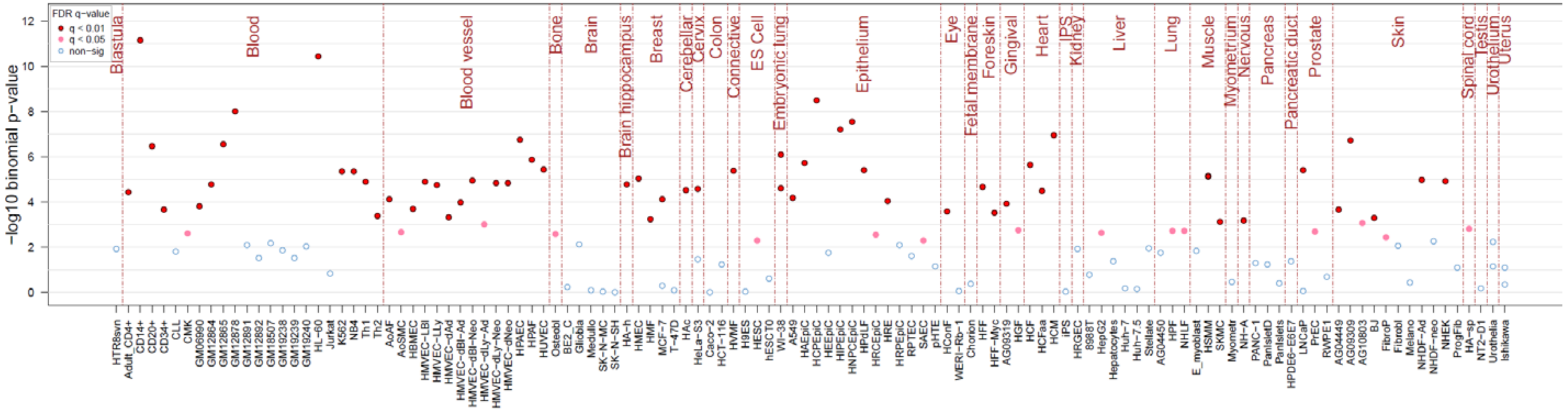

Blueprint Epigenomics – DNAse Hypersensitivity Sites

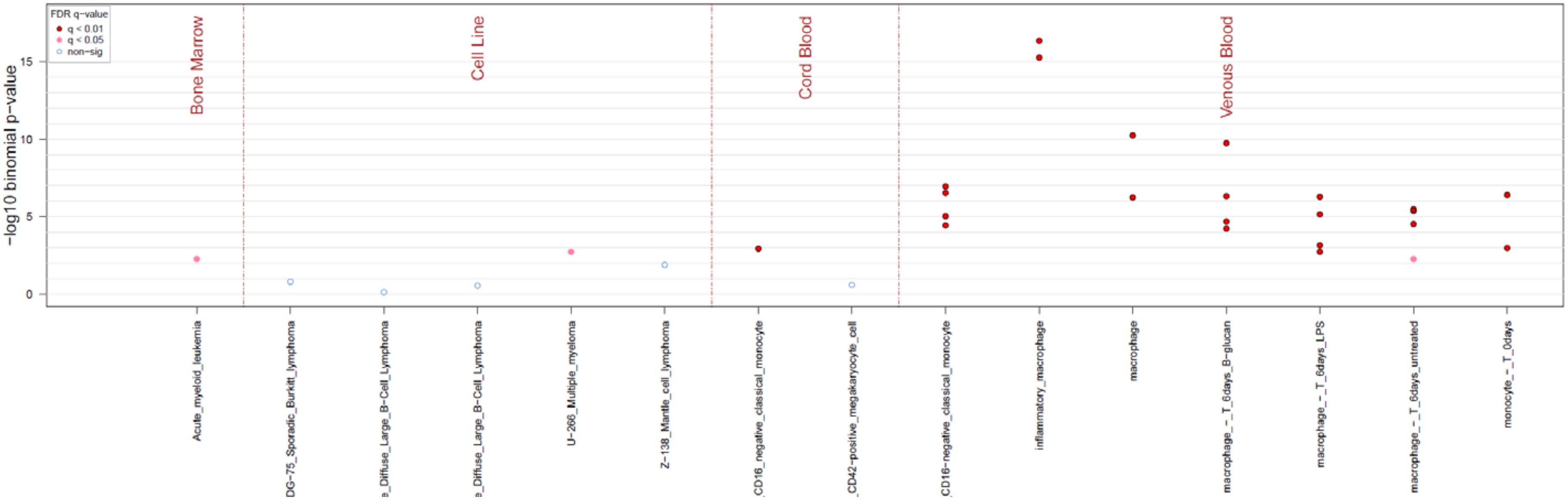

Supplement: Supplementary file 2 [file Image_1.pdf]
